# Canine infection with *Dirofilaria immitis*, *Borrelia burgdorferi*, *Anaplasma* spp., and *Ehrlichia* spp. in the United States, 2010–2012

**DOI:** 10.1186/1756-3305-7-257

**Published:** 2014-05-30

**Authors:** Susan E Little, Melissa J Beall, Dwight D Bowman, Ramaswamy Chandrashekar, John Stamaris

**Affiliations:** 1 Department of Veterinary Pathobiology, Center for Veterinary Health Sciences, Oklahoma State University, Room 250 McElroy Hall, Stillwater, OK 74078, USA; 2 IDEXX Laboratories, Inc., Westbrook, ME, USA; 3 Department of Microbiology and Immunology, College of Veterinary Medicine, Cornell University, Ithaca, NY, USA

**Keywords:** *Anaplasma*, *Borrelia burgdorferi*, Canine, *Dirofilaria immitis*, *Ehrlichia*

## Abstract

**Background:**

The geographic distribution of canine infection with vector-borne disease agents in the United States appears to be expanding.

**Methods:**

To provide an updated assessment of geographic trends in canine infection with *Dirofilaria immitis*, *Borrelia burgdorferi*, *Ehrlichia* spp., and *Anaplasma* spp., we evaluated results from an average of 3,588,477 dogs tested annually by veterinarians throughout the United States from 2010 – 2012.

**Results:**

As in an earlier summary report, the percent positive test results varied by agent and region, with antigen of *D. immitis* and antibody to *Ehrlichia* spp. most commonly identified in the Southeast (2.9% and 3.2%, respectively) and antibody to both *B. burgdorferi* and *Anaplasma* spp. most commonly identified in the Northeast (13.3% and 7.1%, respectively) and upper Midwest (4.4% and 3.9%, respectively). Percent positive test results for *D. immitis* antigen were lower in every region considered, including in the Southeast, than previously reported. Percent positive test results for antibodies to *B. burgdorferi* and *Ehrlichia* spp. were higher nationally than previously reported, and, for antibodies to *Anaplasma* spp., were higher in the Northeast but lower in the Midwest and West, than in the initial report. Annual reports of human cases of Lyme disease, ehrlichiosis, and anaplasmosis were associated with percent positive canine test results by state for each respective tick-borne disease agent (R^2^ = 0.701, 0.457, and 0.314, respectively). Within endemic areas, percent positive test results for all three tick-borne agents demonstrated evidence of geographic expansion.

**Conclusions:**

Continued national monitoring of canine test results for vector-borne zoonotic agents is an important tool for accurately mapping the geographic distribution of these agents, and greatly aids our understanding of the veterinary and public health threats they pose.

## Background

In 2009, we reported results of a national veterinary clinic based survey of dogs in the United States for antigen to heartworm and antibody to tick-borne disease agents [1]. Based on reported results from testing over 3 million dogs from 2001 to 2007, this study was the first to document and map percent positive test results on a national level to four vector-borne disease agents, namely, *Dirofilaria immitis*, *Borrelia burgdorferi*, *Anaplasma* spp., and *Ehrlichia* spp. [1]. *Dirofilaria immitis*, the causative agent of heartworm disease, is transmitted to dogs by a number of different mosquito species and is present throughout much of the United States [2]. *Borrelia burgdorferi* and *Anaplasma phagocytophilum* are transmitted by *Ixodes* spp. ticks, while *Ehrlichia* spp. are known to be vectored by *Rhipicephalus sanguineus* sensu lato as well as ticks in the genera *Amblyomma*, *Dermacentor*, and *Ixodes*[3,4].

Veterinarians and pet owners are aware of these infections, and canine preventive medicine protocols commonly include recommendations for administering routine heartworm prevention and tick control to dogs as well as vaccination to prevent transmission of *B. burgdorferi*, the agent of Lyme disease, in areas where transmission occurs [5]. However, not all dogs receive adequate veterinary care, prevention recommendations are not consistently followed, and even when implemented, these strategies are not 100% effective at preventing infection [2,6,7].

Because vector-borne infections can have serious implications for canine health, annual testing of dogs for these infections is recommended and commonly performed [2,5,8]. A number of studies have confirmed the utility of dogs as sentinels for tick-borne diseases [9-12]. Indeed, our original report has been widely cited and the data repurposed by other research groups [13], and subsequent work has shown that canine infection with some tick-borne disease agents correlates on a state-wide basis with reports of human disease [14]. Here, we provide an update to our original report by summarizing the percent positive test results of dogs tested by veterinarians in the United States from 2010 – 2012.

## Methods

### Source of data

Testing results included in the 2010–2012 summary and analysis were obtained from 5 different USDA licensed test kits manufactured by IDEXX Laboratories, Inc: PetChek® Heartworm PF Test, a microtiter plate ELISA for use in-clinic or at a reference laboratory for the detection of *D. immitis* antigen in canine serum or plasma; SNAP® HW RT Test kit, an in-clinic ELISA for the detection of *D. immitis* antigen in canine serum, plasma, or whole blood; SNAP® 4Dx® Test kit, an in-clinic ELISA for simultaneous detection of canine antibodies to *E. canis, B. burgdorferi* and *A. phagocytophilum*, and to *D. immitis* antigen; and SNAP® 4Dx® Plus Test kit, which was released in 2012 to replace SNAP 4Dx and is an in-clinic ELISA for simultaneous detection of canine antibodies to *E. canis, E. ewingii*, *B. burgdorferi, A. phagocytophilum*, and *A. platys,* and to *D. immitis* antigen.

Results of testing on these various test kits were obtained from two primary sources: the IDEXX Reference Laboratories network (PetChek® Heartworm PF, SNAP® 4Dx®, and SNAP® 4Dx Plus®), and those results generated by veterinarians using all five assays and recorded in IDEXX VetLab® Instrumentation and Software. For the latter results, information was obtained for both results entered manually by the clinic staff and those automatically recorded by IDEXX SnapShot Dx® instrumentation. For reasons of privacy, patient results were obtained in the absence of owner information or unique identification. Because of this, it was not possible to exclude repeat testing events either within a practice or between the practice and the reference laboratory.

### Performance of test kits

Performance of the PetChek® Heartworm PF Test, SNAP® HW RT Test kit, SNAP® 3Dx® Test kit, and SNAP® 4Dx® Test kit has been reported previously [1,15]. The SNAP® 4Dx Plus® Test uses a peptide from a major outer surface protein (p28) of *E. ewingii* on the *Ehrlichia* portion of the test and has 96.5% sensitivity and 93.9% specificity for the detection of *E. ewingii* antibodies. The *Anaplasma* portion of the SNAP® 4Dx Plus® Test uses a synthetic peptide from the major surface protein of *A. phagocytophilum* (MSP2/p44) and has 89.1% sensitivity and 99.8% specificity for the detection of *A. platys* antibodies [16].

### Data and statistical analysis

Test results were compiled by county based on the associated postal zip code of the veterinary hospital submitting the sample or providing the test result. Data were assembled into state and regional groups as previously described [1,17]. Four primary regional groups (Midwest, Northeast, Southeast, and West) were considered. Percent positive results were calculated by dividing the number of tests reported as positive for each agent by the total number of testing events recorded in a given county, state, or region. For state-wide summary tables and comparison to human disease reports, all results collected from 2010 – 2012 were included. For construction of county-based prevalence maps, individual counties in which fewer than 30 test results were available from a single year were excluded. Differences in the frequency of reported positive test results between counties, states, and regions, as well as differences in frequency of reported positive test results in the present survey and in our earlier report [1], were evaluated for significance with Chi-square test using SAS (Windows 9.1) (SAS Institute Inc., Cary, NC) with significance assigned at p < 0.0001.

Human cases of Lyme disease, ehrlichiosis, and anaplasmosis reported to the Centers for Disease Control and Prevention in 2010 and 2011 [18] were adjusted to reflect reported cases per 100,000 using average state population data based on intercensal estimates from the United States Census Bureau [19]; summaries of reported human cases from 2012 are not yet available. Comparison of population adjusted reports of human disease to canine seroprevalence rates for each respective agent (*B. burgdorferi*, *Ehrlichia* spp., and *Anaplasma* spp.) were performed using a linear regression and the coefficient of determination (R^2^) calculated as previously described [14]. Analyses were performed using GraphPad Prism v.5 (GraphPad Software, La Jolla, CA).

## Results

### Summary

**Figure 1:**
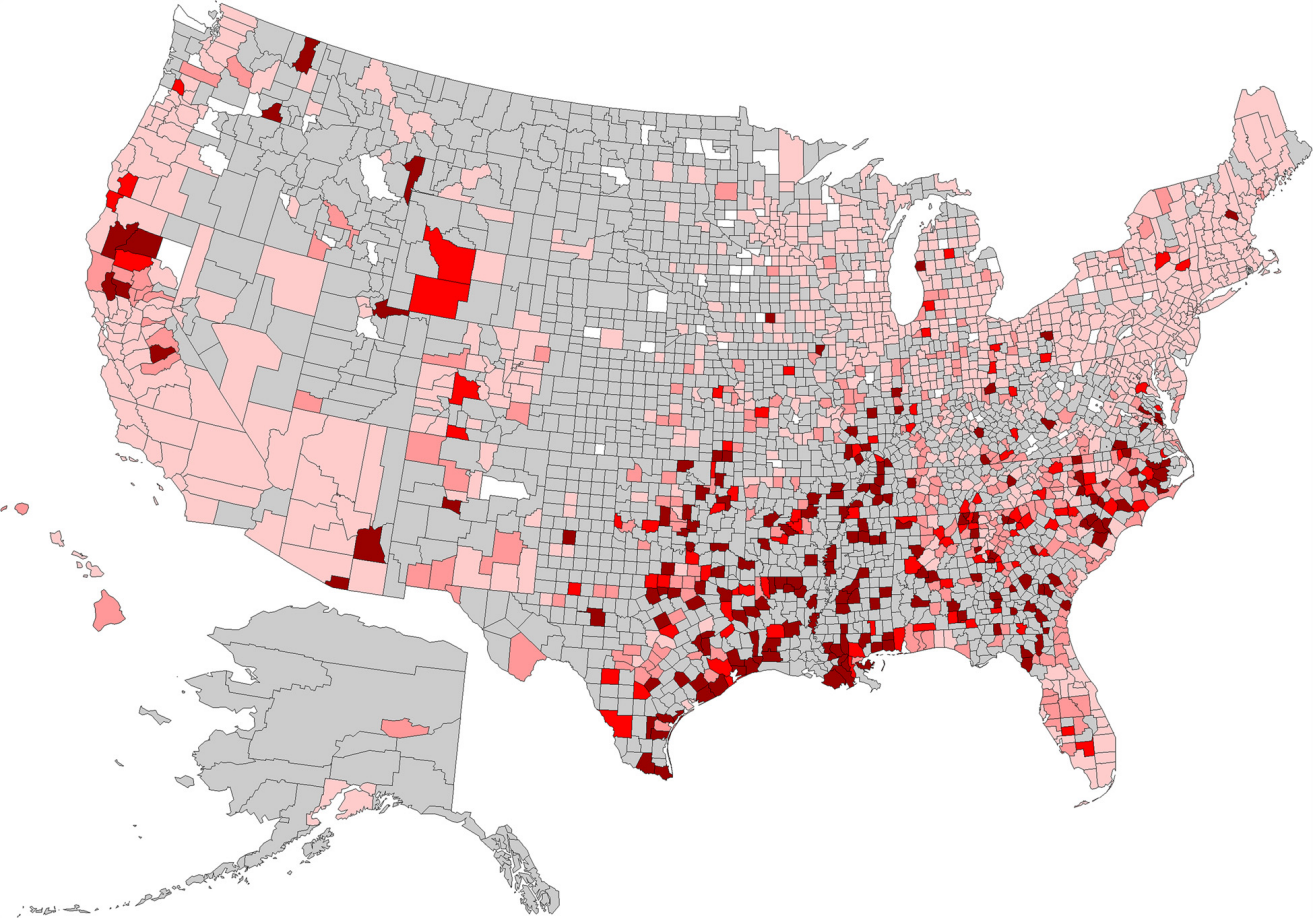
**Evidence of antigen to *Dirofilaria immitis *in dogs by county, grouped according to percent positive tests.** No results (<30) were received from counties shaded gray, precluding interpretation of the presence of antigen in dogs from these areas. Counties depicted in white had no dogs reported as positive (0%). Remaining counties were coded as follows: 0.1-2.0% (light pink), 2.1-4.0% (pink), 4.1-6.0% (red), and > 6.0% (dark red).

**Figure 2:**
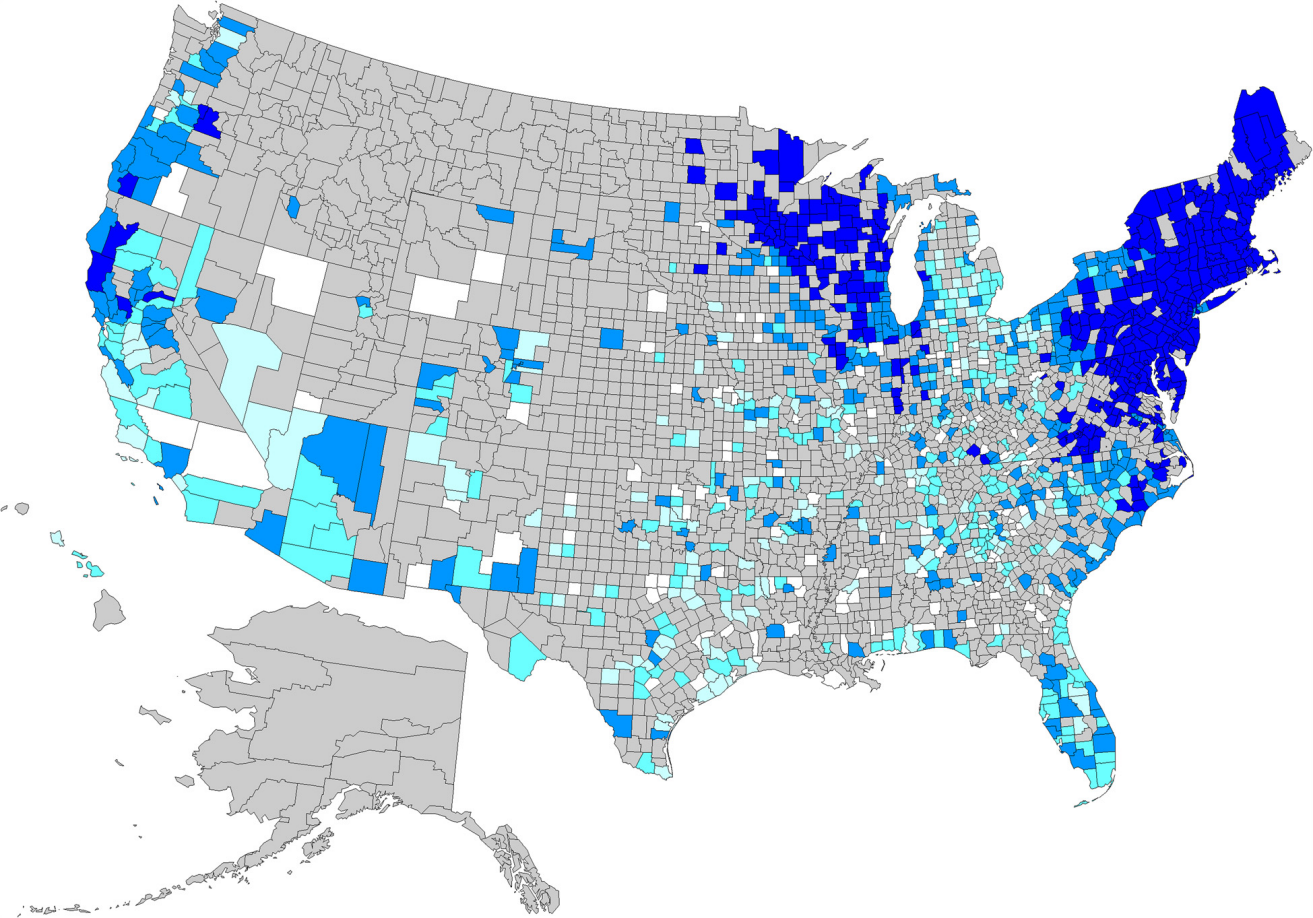
**Evidence of antibody to *Borrelia burgdorferi *in dogs by county, grouped according to percent positive tests.** No results (<30) were received from counties shaded gray, precluding interpretation of the presence of antibody in dogs from these areas. Counties depicted in white had no dogs reported as positive (0%). Remaining counties were coded as follows: 0.1-0.5% (light blue), 0.5-1.0% (blue), 1.1-5.0% (dark blue), and > 5.0% (very dark blue).

**Figure 3:**
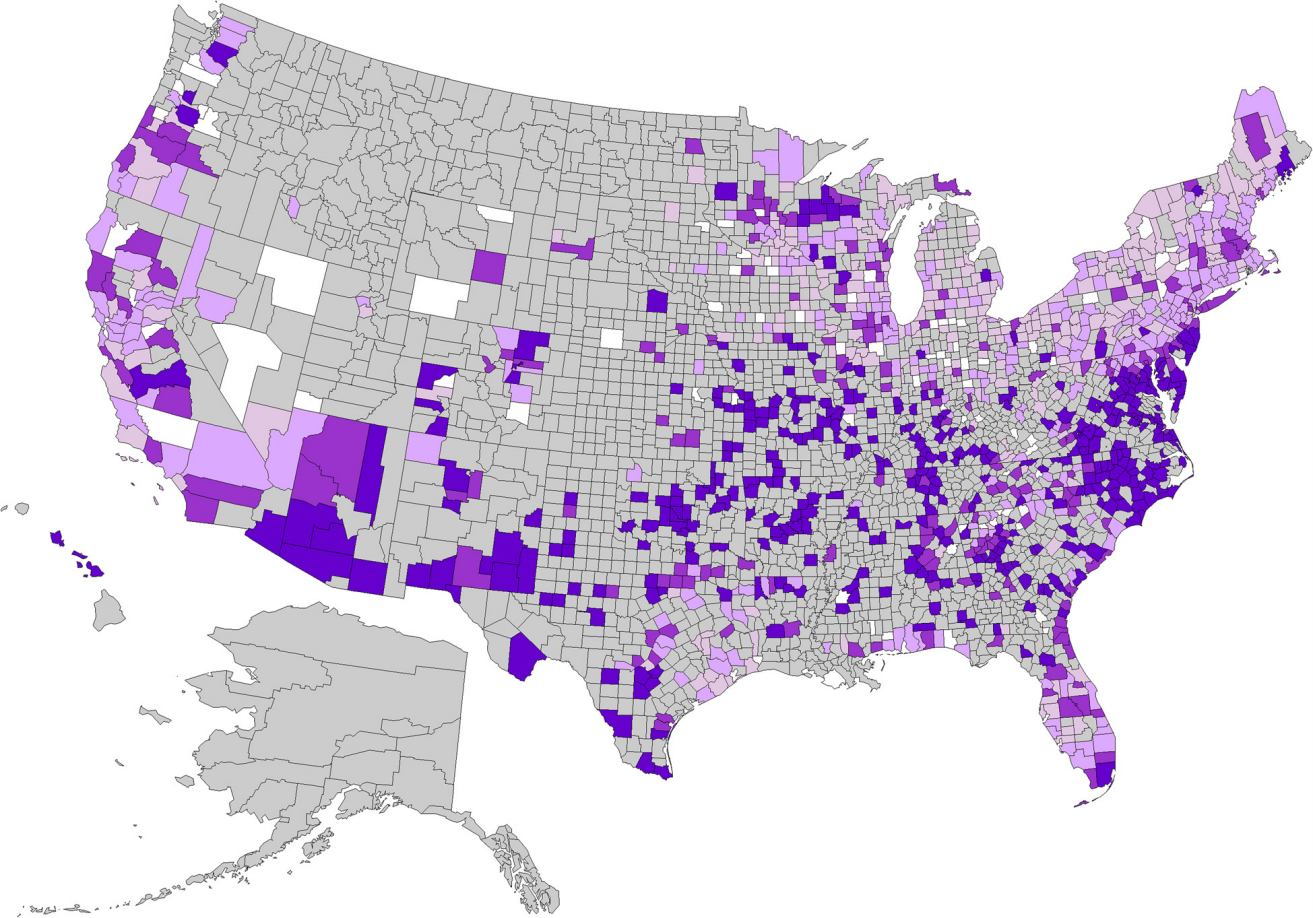
**Evidence of antibody to *Ehrlichia *spp. in dogs by county, grouped according to percent positive tests.** No results (<30) were received from counties shaded gray, precluding interpretation of the presence of antibody in dogs from these areas. Counties depicted in white had no dogs reported as positive (0%). Remaining counties were coded as follows: 0.1-0.5% (light purple), 0.5-1.0% (purple), 1.1-2.0% (dark purple), and > 2.0% (very dark purple).

**Figure 4:**
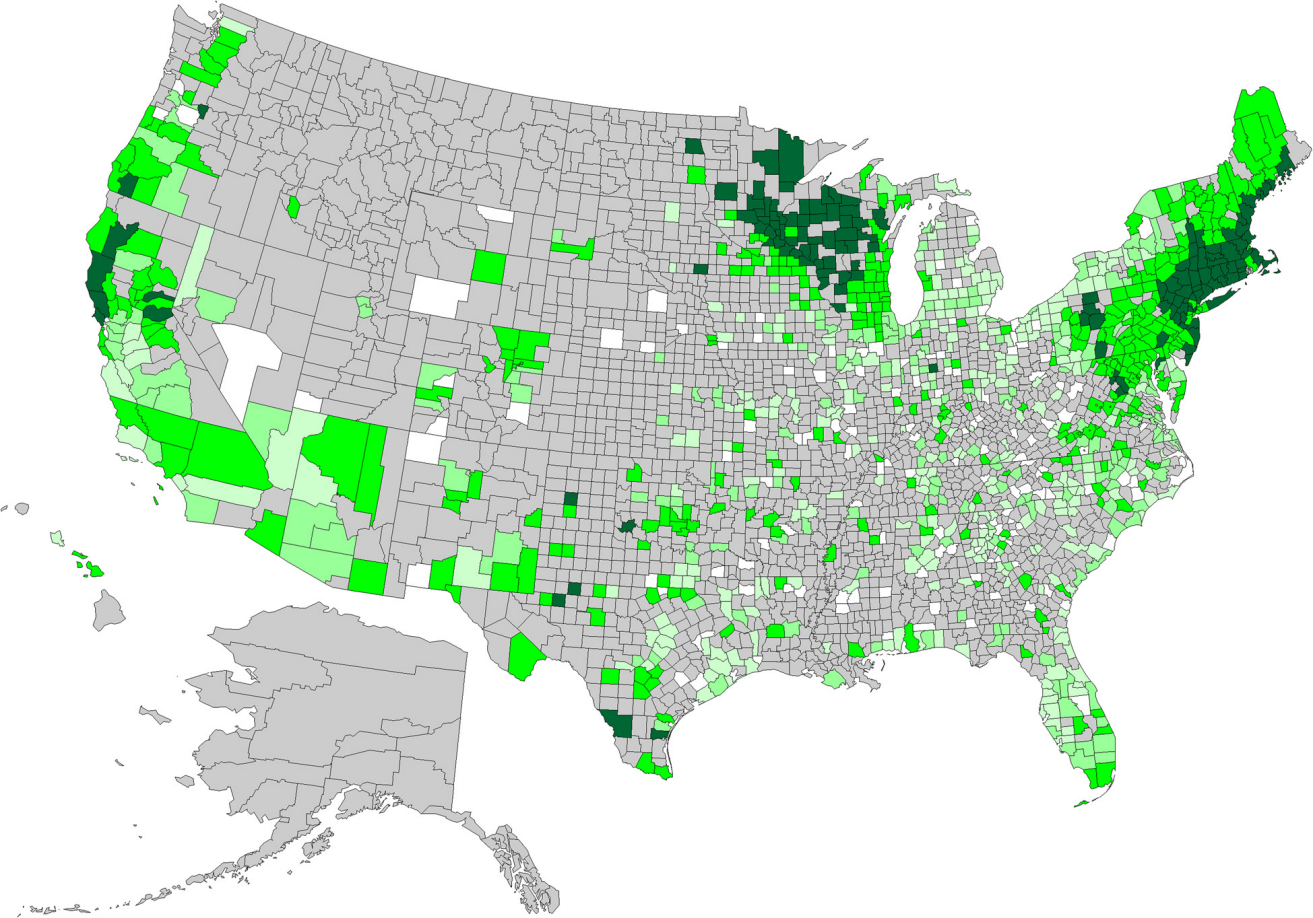
**Evidence of antibody to *Anaplasma *spp. in dogs by county, grouped according to percent positive tests.** No results (<30) were received from counties shaded gray, precluding interpretation of the presence of antibody in dogs from these areas. Counties depicted in white had no dogs reported as positive (0%). Remaining counties were coded as follows: 0.1-0.5% (light green), 0.5-1.0% (green), 1.1-5.0% (dark green), and > 5.0% (very dark green).

A total of 30,917,280 data points were available from dogs tested in 1,778 counties and in all of the 50 states in the United States over the three year period summarized in the present paper (Table 1); evidence of at least one agent was found in dogs from every state considered. Antigen to heartworm was identified in dogs in every state, and antibodies to the tick-borne agents were identified in dogs in every state except Alaska, where test results were not available, and, for *B. burgdorferi* and *Ehrlichia* spp., Montana. Distribution of positive tests and relative percent positive values by county and state are shown in Figures 1, 2, 3 and 4.

**Table 1:** **Percent positive test results (number positive/number tested) by region for dogs tested from 2010 – 2012 in the United States for antigen of *Dirofliaria immitis *** **and antibody to *Borrelia burgdorferi*, *Ehrlichia *** **spp., and *Anaplasma *** **spp.**

**State**	***Dirofilaria immitis***	***Borrelia burgdorferi***	***Ehrlichia *** **spp.**	***Anaplasma *** **spp.**
Northeast	0.4% (11,675/3,175,080)	13.3% (373,212/2,806,273)	0.9% (24,011/2,806,112)	7.1% (189,486/2,652,801)
Midwest	0.7% (20,014/2,817,915)	4.4% (76,025/1,720,510)	1.0% (17,337/1,720,168)	3.9% (59,580/1,548,686)
Southeast	2.9% (101,850/3,562,190)	2.5% (51,232/2,058,574)	3.2% (65,191/2.057,984)	0.9% (14,046/1,631,332)
West	0.8% (8,887/1,178,947)	1.4% (5,726/410,840)	1.3% (5,134/410,419)	2.0% (7,056/359,449)
**Overall mean**	**1.3% (142,426/10,734,132)**	**7.2% (509,195/6,996,197)**	**1.6% (111,673/6,994,683)**	**4.4% (270,168/6,192,268)**

### Heartworm

Percent positive test results for antigen of *D. immitis* were higher in the Southeast than in the other three regions, and were higher in the West and Midwest than in the Northeast (Table 1). Within areas of relatively low percent positive test results, certain counties had unexpectedly high results, including Belknap County, New Hampshire; Summit County, Utah; Walla Walla and Stevens Counties in Washington; and Shasta and Trinity Counties in California (Figure 1). Regional prevalence of percent positive test results for *D. immitis* antigen was lower in each of the four regions considered, and dramatically so in the Southeast, as compared to our previous report.

### Lyme disease

Percent positive test results for antibody to *B. burgdorferi* were higher in the Northeast than in the other three regions, and were higher in the Midwest than in the Southeast or West (Table 1). Omitting Virginia and West Virginia (9.7% and 3.5%, respectively), where Lyme borreliosis is known to be endemic, from the Southeast resulted in a percent positive test result of 0.92% (15,086/1,631,562) in the remainder of the region. Within areas of relatively low percent positive test results, certain counties had unexpectedly high results, including Pulaski and Whitley Counties in Kentucky; Monroe County in Indiana; and Clark, Crawford, and Lawrence Counties in Illinois (Figure 2). Regional prevalence of percent positive test results for *B. burgdorferi* antibody was higher in the Northeast, Southeast, and Midwest, but unchanged in the West, compared to our previous report.

### Ehrlichiosis

Percent positive test results for antibody to *Ehrlichia* spp. were higher in the Southeast than in the other three regions, and were higher in the West than in the Northeast or Midwest (Table 1). Within areas of relatively low percent positive test results, certain counties had unexpectedly high results, including Douglas and La Plata Counties in Colorado; Hancock County, Maine; Clackamas County, Oregon; Lamoille County, Vermont; and Clark and King Counties in Washington (Figure 3). Percent positive test results for *Ehrlichia* spp. antibody were higher nationally and in every region considered compared to our previous report.

### Anaplasmosis

Percent positive test results for antibody to *Anaplasma* spp. were highest in the Northeast and Midwest, and were higher in the West than in the Southeast (Table 1). Within areas of relatively low percent positive test results, certain counties had unexpectedly high results, including Jackson County, Oklahoma; Howard and Potter Counties in Texas; and Minnehaha County, South Dakota (Figure 4). Percent positive test results for *Anaplasma* spp. antibody were lower nationally and in the Midwest and West, but higher in the Northeast, compared to our previous report.

### Comparison to human disease reports

Percent positive test results for antibodies to *B. burgdorferi* in dogs and reported human cases of Lyme borreliosis by state were positively associated (R^2^ = 0.701, *F* = 110.0), although for some states reported human cases were higher (Delaware and New Hampshire) or lower (Rhode Island and South Dakota) than expected based on canine testing. Percent positive test results for *Ehrlichia* spp. in dogs and human case reports of ehrlichiosis by state were positively associated (R^2^ = 0.457, *F* = 34.5), although for some states reported human cases were higher (Oklahoma, Missouri, and Delaware) or lower (Arizona, Mississippi, and Washington) than expected based on canine testing. Percent positive test results for *Anaplasma* spp. in dogs and human case reports of anaplasmosis by state were positively associated (R^2^ = 0.314, *F* = 18.8), although for some states reported human cases were higher (Minnesota and Wisconsin) or lower (Connecticut and South Dakota) than expected based on canine testing.

## Discussion

Vector-borne infections are increasingly important to the health of people and other animals worldwide [20-22]. The data reported in the present paper update our previous study and comprise the most comprehensive effort to date to document the distribution and prevalence of infection with these agents using canine samples [1]. Annual testing for heartworm and tick-borne infections is recommended and commonly performed by veterinarians [2,5]. We are not able to identify individual dogs or eliminate test results from the same dogs with the reporting system used, and some of the results undoubtedly reflect repeated testing of the same dogs. However, even if the results reported here represent repeated testing of 3 – 4 million pet dogs, we have likely documented the past or current infection status of approximately 5% of the estimated 70 million pet dogs in the United States, and perhaps as many as 10 – 15% [23].

Test results were available from at least 30 dogs from 1,420 counties, constituting 45.2% of the 3,144 counties in the United States. Because dogs cluster in human population centers, the present paper is biased towards urban areas, an approach which may underestimate true infection risk in some locales, particularly for tick-borne infections. Although urban transmission patterns have been described, the risk of tick-borne disease is generally greater in areas of lower housing density with more supportive habitat for the vector ticks, [24-27]. Moreover, an adequate number of test results were not available from many sparsely populated regions and thus we cannot determine the likelihood for these infections to be present across the entire nation.

As expected, heartworm infection was most commonly identified in dogs in the southeastern United States although the percent positive test results in this region were lower than previously reported [1]. While we cannot fully explain this reduction, the decrease may have been due to differences in data capture strategies, which resulted in inclusion of a higher frequency of routine heartworm tests in the data set. Strains of *D. immitis* resistant to heartworm preventives have recently been described from the southern United States, and veterinarians may be responding with greater vigilance [28-30]. Repeatedly testing dogs on heartworm preventive, and thus at low risk of infection, would be expected to decrease the percent positive test results overall. However, several states in the area of the country most affected by reports of resistance, including Alabama, Georgia, Louisiana, and Mississippi, actually have higher percent positive test results in the present paper than were reported previously [1], (Table 1).

Although the national prevalence of positive tests for heartworm in the present study was lower, the overall geographic distribution of positive test results was similar to that described in the previous report. Within this expected pattern, a number of counties with unexpectedly high percent positive test results were identified. Some of these unexpected results can be explained by the relatively low number (n = 30–45, data not shown) of test results available. Testing only a small number of dogs has been shown to result in an inaccurate impression of the presence of vector-borne transmission cycles due to translocation of infected pets [31]. In contrast, the counties identified with high percent positive test results in northern California were based on testing thousands of dogs and are consistent with our current understanding of *D. immitis* prevalence in domestic dogs and wild canids in that region [32,33].

In contrast to heartworm, percent positive results for antibody to agents of tick-borne diseases, particularly Lyme disease and ehrlichiosis, were higher nationally in the present study than in the previous report [1], a finding consistent with increased reports of these diseases in people in recent years [34,35]. Increases in percent positive test results were also seen regionally with the most striking change in the Southeast, where a nearly 1.5 fold increase in prevalence of *B. burgdorferi* was seen, largely due to increases in the percent positive test results of dogs in the states of Virginia, West Virginia, and Kentucky. A similar increased geographic distribution of the risk of *B. burgdorferi* infection has been described in public health reports although autochthonous transmission of this infection in people appears to remain focused in clear endemic and hyperendemic regions [34,36].

An increase in percent positive test results to *Ehrlichia* spp. was also evident in each of the four regions (Table 1). This increase was not attributable to recent modifications in the assay to include detection of antibodies to *E. ewingii*[14,37] because it was evident in the data in 2010 and 2011 (data not shown), before any platform changes were instituted. Moreover, the more widespread geographic distribution parallels reports from human surveillance and suggests the distribution of autochthonous transmission has increased, consistent with other data documenting continued geographic expansion of *A. americanum* ticks [35,38-41]. However, we suspect infection with a novel *Ehrlichia* sp. transmitted by ticks other than *A. americanum* are responsible for the positive test results that continue to be identified in Minnesota and Wisconsin, as has been previously described [1,42,43].

The percent positive test results for *Anaplasma* spp. were lower overall, although higher in the Northeast, than in the previous report, although large geographic variation was seen within the regions considered. Reports of human anaplasmosis cases have also increased in the Northeast in recent years [35,44]. The foci of elevated percent positive test results identified in dogs in western Texas and Oklahoma are mostly likely due to *R. sanguineus* transmitted infection with *A. platys*[14,45]. Brown dog ticks are common in this region, remarkably tolerant of arid conditions, and thrive during the 2011–2012 drought in the southcentral United States [46], Little, unpubl. data.

For each of the tick-borne agents, a few counties were identified with unexpectedly high percent positive test results given their geographic location within areas largely considered low or non endemic for those diseases. Some of these unexpected results are likely due to the low number of test results available (<300), while others may be due to the presence of novel agents. While the overall pattern in the maps provided is of great value in understanding distribution of disease, results for individual counties, particularly in areas not likely to support autochthonous transmission based on known vector phenology, should be interpreted with caution [1]. In addition, some of the assays used may detect novel agents as previously described [1,42,43]. Indeed, infection with an *Ehrlichia* sp. other than *E. canis*, *E. chaffeensis*, or *E. ewingii* may account for the higher than expected percent positive test results identified Clark and King Counties in Washington, where results from more than 2,000 dogs were available (Figure 3).

The canine serology results for the tick-borne disease agents in the present study largely agreed with reports of human disease for each respective type of disease. However, the association was strongest for canine antibodies to *Borrelia burgdorferi* and human Lyme disease, a finding which is not surprising as the assay used in dogs is known to be exquisitely specific and canine serology has been shown previously to correspond with reports of Lyme borreliosis in people [1,13,47]. The associations between canine serology and reports of human ehrlichiosis and anaplasmosis, while significant, were admittedly weak. The assays used in the present study detect multiple *Ehrlichia* spp. and *Anaplasma* spp., including some agents thought unlikely to cause disease in people, such as *E. canis* and *A. platys*, suggesting canine seroprevalence data may be of limited value in understanding the distribution of and transmission risk for those zoonotic agents [47-49]. Previous studies which showed strong agreement between canine seroprevalence to *Ehrlichia* spp. and reports of human ehrlichiosis employed species-specific serologic assays, a strategy which may be necessary to generate accurate predictive models for zoonotic infection risk [14]. Nonetheless, there were geographies with discordant results for human ehrlichiosis even using specific canine assays. This discrepancy may be, in part, due to the lack of species-specific diagnostic tools for use in human medicine when detecting antibodies to *E. chaffeensis* and *A. phagocytophilum*[50,51].

Annual canine preventive medicine protocols include testing for evidence of vector-borne infections in addition to implementing preventive strategies like routine acaricide use and vaccination. The canine serology results help veterinarians to assess the efficacy of the prevention protocol for that particular patient and modify it accordingly if evidence of transmission is identified [52]. By testing all dogs within a practice, it is possible to gain a broader perspective on the risks of transmission for recognized disease agents as well as identify the emergence of a previously uncommon pathogen. Translocation of infected dogs also occurs [1,31], complicating interpretation, although identifying infection with disease agents outside the range where they are known to be transmitted can be important to the health of individual patients. Finally, by understanding the relative importance of different vector-borne diseases within their practice area, veterinarians provide a public health service to the community by educating pet owners not only on the risks for their dog but the potential risk that these vector-borne infections may have for public health.

## Conclusions

In this study, we have provided a comprehensive update on the frequency of positive test results for the most common vector-borne disease agents in the dog in the United States. While the broad geographical trends remained consistent with the previous report, several important differences were noted. Positive test results for *D. immitis* antigen were significantly lower in every region considered, while percent positive test results for antibodies to *B. burgdorferi* and *Ehrlichia* spp. were higher nationally than previously reported, and antibodies to *Anaplasma* spp. were higher in the Northeast but lower in the Midwest and West. Canine serology and the frequency of positive test results is an important tool for accurately mapping the geographic distribution of these agents. Recognizing geographic variations in percent positive test results also raises opportunities for future investigations on the distribution of tick species and the risk of human tick-borne disease in both endemic and non-endemic areas.

## Competing interests

In the past five years, SL and DB have received reimbursement, speaking fees, or research support from IDEXX Laboratories, manufacturer of diagnostic tests for heartworm and tick-borne disease agents. In addition, MB, RC, and JS are employees of IDEXX Laboratories.

## Authors’ contributions

SL, DD, and MB conceived of the study, SL and MB coordinated its design and execution and drafted the manuscript, and DD, RC, and JS reviewed and validated the data and the manuscript. All authors read and approved the final version of the manuscript.
